# LncRNA Expression in CD4+ T Cells in Neurosyphilis Patients

**DOI:** 10.3389/fcimb.2017.00461

**Published:** 2017-11-08

**Authors:** Li-Li Liu, Shao-Gang Zhu, Xiao-Yong Jiang, Jun Ren, Yong Lin, Ning-Ning Zhang, Man-Li Tong, Hui-Lin Zhang, Wei-Hong Zheng, Hua-Jun Fu, Hai-Juan Luo, Li-Rong Lin, Jiang-Hua Yan, Tian-Ci Yang

**Affiliations:** ^1^Center of Clinical Laboratory, Zhongshan Hospital, Medical College of Xiamen University, Xiamen, China; ^2^Department of Dermatology, Zhongshan Hospital, Medical College of Xiamen University, Xiamen, China; ^3^Department of Neurology, Zhongshan Hospital, Medical College of Xiamen University, Xiamen, China; ^4^Cancer Research Center, Medical College of Xiamen University, Xiamen, China

**Keywords:** lncRNA, CD4-Positive T-Lymphocytes, neurosyphilis, treponema pallidum, microarray

## Abstract

Recent studies have shown that several long noncoding RNAs (lncRNAs) are involved in regulating the immune response to cope with pathogenic invasion. To date, the roles of lncRNAs in the CD4+ T cell response to *Treponema pallidum* (*T. pallidum*) infection in neurosyphilis patients remain unknown. The mRNA and lncRNA expression profiles of CD4+ T cells that were isolated from neurosyphilis patients and healthy controls were analyzed by microarray. A total of 2258 lncRNAs and 1728 mRNAs were identified as over-expressed or under-expressed, respectively (fold change > 1.5) in the CD4+ T cells of neurosyphilis patients compared to the healthy controls. The lncRNA-mRNA co-expression network showed that 59 lncRNAs showed significant differences along with significantly different mRNAs. Among the 59 gene pairs, the *LOC79999* mRNA was positively correlated with the *RP11-160E2.16, RP11-160E2.11*, and *RP11-160E2.19* lncRNAs, and the *NKX1-1* mRNA was positively correlated with the *RP11-1398P2.1, RP11-160E2.19*, and *XLOC_003422* lncRNAs. The following five mRNAs were correlated with two differential lncRNAs: *DUSP16, AP000349.1, FAM115C, TIMM8A*, and *SMCHD1*. Gene Ontology (GO) analysis revealed that the differentially expressed coding genes were mainly involved in biological processes and the top 4 terms that associated with above-mentioned differentially expressed coding genes were as follows: defense response to fungus, defense response to bacterium, killing of cells of other organism and disruption of cells of another organism. A subsequent pathway analysis was also conducted, and several pathways, including the T cell receptor, MAPK, and TGF-beta signaling pathways, were associated with the differentially expressed mRNAs. This study reveals the differential expression profiles of lncRNAs in the CD4+ T cell response to the *T. pallidum* infection in neurosyphilis patients. LncRNAs are involved in key biological processes that comprise the CD4+ T cell response to the *T. pallidum* infection.

## Introduction

Syphilis is a chronic systemic sexually transmitted disease that is caused by *Treponema pallidum* (*T. pallidum*) (Tong et al., 2014). Data released by China Centers for Disease Control and Prevention (http://www.nhfpc.gov.cn/) showed that there were 464,457 newly infected syphilis cases in China in 2016, including 65 deaths. Moreover, the syphilis incidence increased by 7.02% over the same period in 2015, and syphilis continued to be the sexually transmitted disease with highest incidence. Neurosyphilis is one of the most disruptive clinical types of syphilis and is difficult to contain. The high prevalence of syphilis has led to a sustained increase in the incidence of neurosyphilis, with ~70% of syphilis patients demonstrating cerebrospinal fluid (CSF) abnormalities(Marra et al., 2004) and 25–40% of untreated or partially treated patients having the possibility of developing neurosyphilis (Zetola et al., 2007). Accordingly, the prevalence of neurosyphilis patients in China was estimated to have reached 1.9 million in 2016. The sources of purified isolates of *T. pallidum* are limited because *T. pallidum* is uncultivable *in vitro* and slowly reproduces *in vivo*. Additionally, the fragile outer membrane of *T. pallidum* and the absence of a target for genetic manipulation have greatly increased the difficulty of performing knock-in experiments, knock-out experiments, silencing analyses, mutagenesis and other genetic alterations in this pathogen (LaFond and Lukehart, 2006; Liu et al., 2014). Currently, knowledge of the occurrence of natural regression and the mechanism of neurological damage after *T. pallidum* infects the central nervous system is limited. In a previous study, we examined the immune functions of 42 neurosyphilis patients and observed that a cellular immune imbalance and a significant reduction in CD4+ T cells occurred in the white blood cells of neurosyphilis patients (Liu et al., 2013a). Recent studies have shown that several long noncoding RNAs (lncRNAs) are involved in regulating the immune response to cope with pathogenic invasion (Zur Bruegge et al., 2017). LncRNAs have a length more than 200 nt, occupy a large proportion of transcriptomes and form a major class of RNA transcripts (Djebali et al., 2012). With the widespread application of next-generation sequencing (NGS) technology, an increasing number of noncoding RNAs have been detected in the human genome (Veneziano et al., 2016). Moreover, studies have confirmed that lncRNA can participate in X chromosome silencing, genomic imprinting, chromatin modification, transcriptional activation, transcriptional interference, intranuclear transport and other important regulatory processes (Yang et al., 2014). Compared with other transcript types, knowledge of lncRNAs (especially in the immune system) is limited. Recently, many lncRNAs in the mammalian genome were shown to be involved in regulating the expression of genes that were associated with innate immune processes (Ouyang et al., 2016). However, unlike protein-coding genes, lncRNAs are poorly conserved, making sequencing-based predictions of lncRNA functions difficult. In the past 2–3 years, progress has been achieved in the study of lncRNA function. For instance, David *et al*. identified the *IFNG-AS1* lncRNA in peripheral blood CD4+ T cells that were acquired from patients with ulcerative colitis. *IFNG-AS1* could regulate the function of inflammatory factor IFNG and serve as a novel biomarker for detecting ulcerative colitis (Padua et al., 2016). Additionally, lncRNA-*IFNG-AS1* was up-regulated in CD4+ T cells that were acquired from patients with Hashimoto's thyroiditis. LncRNA-*IFNG-AS1* can regulate IFNG expression at the transcriptional and translational levels and promote the Th1 immune response (Peng et al., 2015). Li et al. reported that lncRNA-*NRON* in CD4+ T cells could specifically down-regulate the Tat protein and promote the continuous presence of the HIV-1 virus in the body (Li et al., 2016). The endeavors in this field have revealed that lncRNAs can regulate the expression of protein-coding genes by affecting mRNA transcription, splicing and translation and that the physiological processes that are associated with cell proliferation, differentiation and apoptosis would thus be altered (Mattick, 2009).

LncRNAs can regulate the expression of protein-coding genes through different molecular biological mechanisms. Thus, lncRNAs influence disease development by participating in disease-related signaling pathways. Therefore, we hypothesized that *T. pallidum* inhibited CD4+ T cell functions through the regulatory effects of lncRNAs on relevant signaling pathways to consequently promote the development of neurosyphilis.

## Materials and methods

### Human subjects

Twenty neurosyphilis cases from Xiamen Zhongshan Hospital, Medical College of Xiamen University, were enrolled in the study between November 2016 and January 2017. Twenty-three healthy control individuals were recruited from the health examination professionals in Xiamen Zhongshan Hospital. The clinical characteristics are summarized in Table 1. The diagnostic criteria for neurosyphilis complied with the guidelines of the Centers for Disease Control in Europe and America (Janier et al., 2014; Workowski and Bolan, 2015). Neurosyphilis is defined as syphilis at any stage with one or more of the following findings: (1) a reactive RPR in the CSF, (2) an elevated CSF protein concentration (>500 mg/L) and/or leukocyte count (>10 cells/μL) in the absence of other known causes of these abnormalities, and (3) clinical symptoms or signs that are consistent with neurosyphilis without other known causes for these clinical abnormalities.

**Table 1:** Demographic data and clinical data of the patients with neurosyphilis and healthy controls in southern China.

**Clinical parameters**	**Neurosyphilis (*n* = 20)**	**Healthy controls (*n* = 23)**
**Sex**		
Male, n (%)	15 (75.0)	17 (73.9)
Female, n (%)	5 (25.0)	6 (16.1)
Age, median years (range)	58 (21–74)	51 (23–71)
**Origin**		
Local, n (%)	9 (45.0%)	9 (39.1%)
Other city in Fujian Province, n (%)	11 (55.0%)	14 (60.9%)
Other city outside Fujian	0	0
Province, n (%)		
Serum RPR ≥ 1:32, n (%)	3 (15.0%)	0
Serum RPR < 1:32, n (%)	17 (85.0%)	0
Baseline CSF RPR titer, median (IQR)	1:1 (1:1–1:8)	ND
Baseline CSF TPPA, median (IQR)	12.5 (0.36–356.97)	ND
Baseline CSF leukocyte count, median (IQR)	3.5 (1–1122)	ND
>10^*^10^6^/L, n (%)	17 (85.0%)	ND
Baseline CSF protein concentration, median (IQR)	506 (236–1095)	
>500 mg/L, n (%)	19 (95.0%)	ND
HBV positive, n (%)	0	0
HCV positive, n (%)	0	0
HIV positive, n (%)	0	0

*RPR, rapid plasma reagin; CSF, cerebrospinal fluid; TPPA, T. pallidum particle agglutination; IQR, interquartile range; HBV, hepatitis B virus; HCV, hepatitis C virus; ND, not detected*.

Peripheral blood samples were collected from all patients before treatment and from healthy controls.

Participants with infectious diseases were excluded following prior screening for antigens and antibodies to hepatitis B virus (HBV), hepatitis C virus (HCV) and human immunodeficiency virus (HIV)-1 and HIV-2, using enzyme-linked immunosorbent assay (ELISA)-based tests (Beijing Wantai Bio-pharm, Beijing, China) and microbial cultures.

## Ethics statement

This study was carried out in accordance with the recommendations of the Institutional Ethics Committee of Zhongshan Hospital, Medical College of Xiamen University with written informed consent from all subjects. All subjects gave written informed consent in accordance with the national legislation and the Declaration of Helsinki. The protocol was approved by the Institutional Ethics Committee of Zhongshan Hospital, Medical College of Xiamen University.

### Specimens

Blood was collected and divided into three 5-mL tubes that contained EDTA for the microarray assays and 5-mL vacutainer tubes for the serological assays for the RPR, *T. pallidum* particle agglutination(TPPA), HIV, and hepatitis *B*-tests. Lumbar punctures were performed under aseptic conditions. CSF was collected and sent to the clinical laboratory for hematology, chemistry, RPR, and TPPA tests.

### RPR and TPPA assays

The serological and CSF tests for syphilis were performed using RPR (Intec, Xiamen, China) and TPPA (Fujirebio, Tokyo, Japan) assays according to the manufacturer's instructions and our previous studies (Liu et al., 2013b).

### Detection of Anti-HBV and Anti-HIV antibodies

The assessments for the presence of anti-hepatitis B surface antigen (HBsAg), anti-hepatitis B surface antibody (anti-HBs), hepatitis B e antigen (HBeAg), hepatitis B e antibody (anti-HBe), hepatitis B core antigen antibody (anti-HBc) (In Tec, China), and anti-HIV antibody (anti-HIV) were performed using antibody capture ELISAs. Subjects were considered HBV-positive if they tested positive for HBsAg, or HBeAg, or anti-HBe, or anti-HBc (Xiao et al., 2016).

### Isolation of CD4+ T cells

Peripheral blood mononuclear cells (PBMCs) were isolated from whole-blood cells using density centrifugation with Ficoll (Axis-Shield, Oslo, Norge). Then, CD4+ T cells were isolated from PBMCs by negative selection using a human CD4+ T Cell Isolation Kit (Miltenyi Biotec, Bergisch Gladbach, Germany) under the guidance of the manufacturer's instructions. CD4+ T cells were placed in a liquid nitrogen pre-freezing RNase-free vial for 5 min and stored at −78°C prior to RNA extraction. Three samples for each group were used for microarray expression analysis, and all samples were used for real-time quantitative polymerase chain reaction (RT-qPCR).

### RNA extraction and quality control

CD4+ T cells were subjected to RNA extraction using the Trizol reagent (Invitrogen, Carlsbad, CA, USA). The RNA quantity and quality were measured using the NanoDrop ND-1000. RNA integrity was assessed by standard denaturing agarose gel electrophoresis or using the Agilent 2100 Bioanalyzer. For the spectrophotometric analysis, samples were used only if the OD260/OD280 ratio was between 1.8 and 2.1 and the OD260/OD230 ratio was > 1.8. For the electrophoresis analysis, samples were free of genomic DNA contamination, and the 28S/18S ratio of the band intensities was >2.0. Only RNA extracts with total volumes that were higher than 8 μg underwent further analysis.

### Microarray analysis of lncRNA and mRNA expression

Arraystar Human LncRNA Microarray V4.0 is designed for the global profiling of human lncRNAs and protein-coding transcripts. ~40,173 lncRNAs and 20,730 coding transcripts can be detected using the current third-generation lncRNA microarray.

Sample labeling and array hybridization were performed according to the Agilent One-Color Microarray-Based Gene Expression Analysis protocol (Agilent Technology) with minor modifications. Briefly, mRNA samples were purified from total RNA after removal of rRNA (mRNA-ONLY™ Eukaryotic mRNA Isolation Kit, Epicentre). Then, each sample was amplified and transcribed into fluorescent cRNA along the entire length of the transcript without 3′ bias using a random priming method (Arraystar Flash RNA Labeling Kit, Arraystar). The labeled cRNAs were purified using the RNeasy Mini Kit (Qiagen). The concentration and specific activity of the labeled cRNAs (pmol Cy3/μg cRNA) were measured using the NanoDrop ND-1000. One microgram of each labeled cRNA was fragmented by adding 5 μL of 10 × Blocking Agent and 1 μL of 25 × Fragmentation Buffer. The mixture was heated at 60°C for 30 min, and 25 μL of 2 × GE Hybridization Buffer was added to dilute the labeled cRNA. Fifty microliters of the hybridization solution was dispensed into the gasket slide and assembled onto the lncRNA expression microarray slide. The slides were incubated for 17 h at 65°C in an Agilent Hybridization Oven. The hybridized arrays were washed, fixed and scanned using the Agilent DNA Microarray Scanner (part number G2505C).

### RT-qPCR analysis

Total RNA was isolated using Trizol from CD4+ T cells that were isolated from the neurosyphilis patients and normal controls. One microgram of RNA was converted into cDNA using a 20-μL reaction system that contained oligo dT primer and GoScript™ Reverse Transcriptase (Promega Corporation, USA). The primers for the target lncRNAs were designed using NCBI Primer-BLAST, with β-Actin as the endogenous control. The primer sequences were as follows: ENST00000421645, forward primer: 5′-GCCTTGTGTGTTTGCACTCT-3′, reverse primer: 5′-TGGGCACATCTCCATCCAAA-3′; ENST00000429530, forward primer: 5′-TGGCTCAGCTGTAGGACAAAG-3′, reverse primer: 5′-CTGGTTGTTTGCTCCTTCCC-3′; uc002nqf.1, forward primer: 5′- CAGGATCACTCCCTCTGTGC-3′, reverse primer: 5′-CCCAACAAGGGGCATTCAGA-3′; T230820, forward primer: 5′-GAGCAGTGCATCTGTCCTAATATGT-3′, reverse primer: 5′-TAAAACAACGGAGGAAGGAGTGG-3′; β-Actin, forward primer: CTGTGGCATCCACGAAACTA, reverse primer: AGTACTTGCGCTCAGGAGGA. A 50-μL reaction using 2 μL of the template and the SYBR Premix Ex Taq™ II kit (TaKaRa, USA) was run using the ABI PRISM 7500 Real-Time PCR System. A dissociation curve was added to verify a single product. The results were analyzed using the 7500 System SDS software, and the relative expression level was ensured using the 2^−ΔΔCT^method (Zeng et al., 2017).

### Microarray data analysis

The Agilent Feature Extraction software (version 11.0.1.1) was used to analyze the acquired array images. Quantile normalization and subsequent data processing was performed using the GeneSpring GX v12.1 software package (Agilent Technologies). After quantile normalization of the raw data, lncRNAs and mRNAs where at least 3 out of 6 samples had flags in Present or Marginal (“All Targets Value”) were chosen for further data analysis. Differentially expressed lncRNAs and mRNAs between the two groups were identified through fold-change filtering. The fold change was calculated the by comparing the normalized lncRNA expression in CD4+ cells between neuronsyphilis patients and control subjects. Differentially expressed lncRNAs and mRNAs with statistical significance between the two groups were identified through *P*-value/FDR filtering. Threshold values of ≥ 1.5-fold change and a Benjamini-Hochberg corrected *P*-value of ≤ 0.05 were employed. Pathway analysis and GO analysis were applied to determine the roles that these differentially expressed mRNAs played in the biological pathways or GO terms. Hierarchical Clustering and combined analysis were performed using in-house scripts. The microarray data were uploaded to the GEO database under accession number GSE103599.

### The creation of the lncRNA-mRNA co-expression network

A lncRNA-mRNA co-expression network was constructed to analyze the correlation between lncRNA and mRNA expression using a Pearson's correlation analysis. Thus, the target mRNAs which are regulated by lncRNAs can be predicted. The lncRNA-mRNA co-expression network was built based on the correlation analysis of the differentially expressed lncRNAs and mRNAs. For each gene pair, a Pearson correlation was estimated. The network was drawn using Cytoscape. In the network analysis, the blue node represents the lncRNA, and the red node represents the mRNA. The blue lines indicates a negative correlation, and the red lines indicate a positive correlation.

Two Coding Potential Assessment Tool (CPAT)/Coding Potential Calculator (CPC) prediction software programs were employed to predict whether the chosen lncRNAs were noncoding (Kong et al., 2007; Wang et al., 2013).

### Functional prediction of lncRNAs

The Gene Ontology (GO) consortium provides structural descriptions of protein functions and are used as a common language for gene annotation in many organisms (Deng et al., 2004). Differentially expressed mRNAs between the neurosyphilis group and healthy control group were involved in the GO and Kyoto encyclopedia of genes and genomes (KEGG, http://www.genome.jp/kegg/) analysis base on GO and the latest KEGG database.

## Results

### Demographic and clinical characteristics of the study subjects

During the observation period, neurosyphilis was diagnosed in 20 HIV-negative patients who exhibited neurological symptoms and were enrolled in this study. Among the enrolled patients, 15 were male, and 5 were female. The median age of the participants was 58 years (ranging from 21 to 74). All subjects underwent reactive baseline serum RPR and serum TPPA tests. Positive serum RPR results that were ≥ 1:32 were found in 3 (15.0%) patients. The median CSF RPR titers were 1:1, and the median baseline CSF TPPA was 12.5. Seventeen (85.0%) and 19 (95.0%) patients had an abnormal baseline CSF leukocyte count and CSF protein concentration, respectively. The control group consisted of 23 healthy subjects, and the serum RPR and TPPA results were negative in the control group as expected. The characteristics of the study participants are summarized in Table 1.

Three patients with neurosyphilis and three healthy subjects were selected for the microarray analysis. Specifically, the peripheral blood was collected, and CD4+ T cells were isolated for further analysis. The expression profiles of the lncRNAs were standardized using the quantile-normalizing method, and the probes that had low-quality measurements were filtered out.

**Figure 1:**
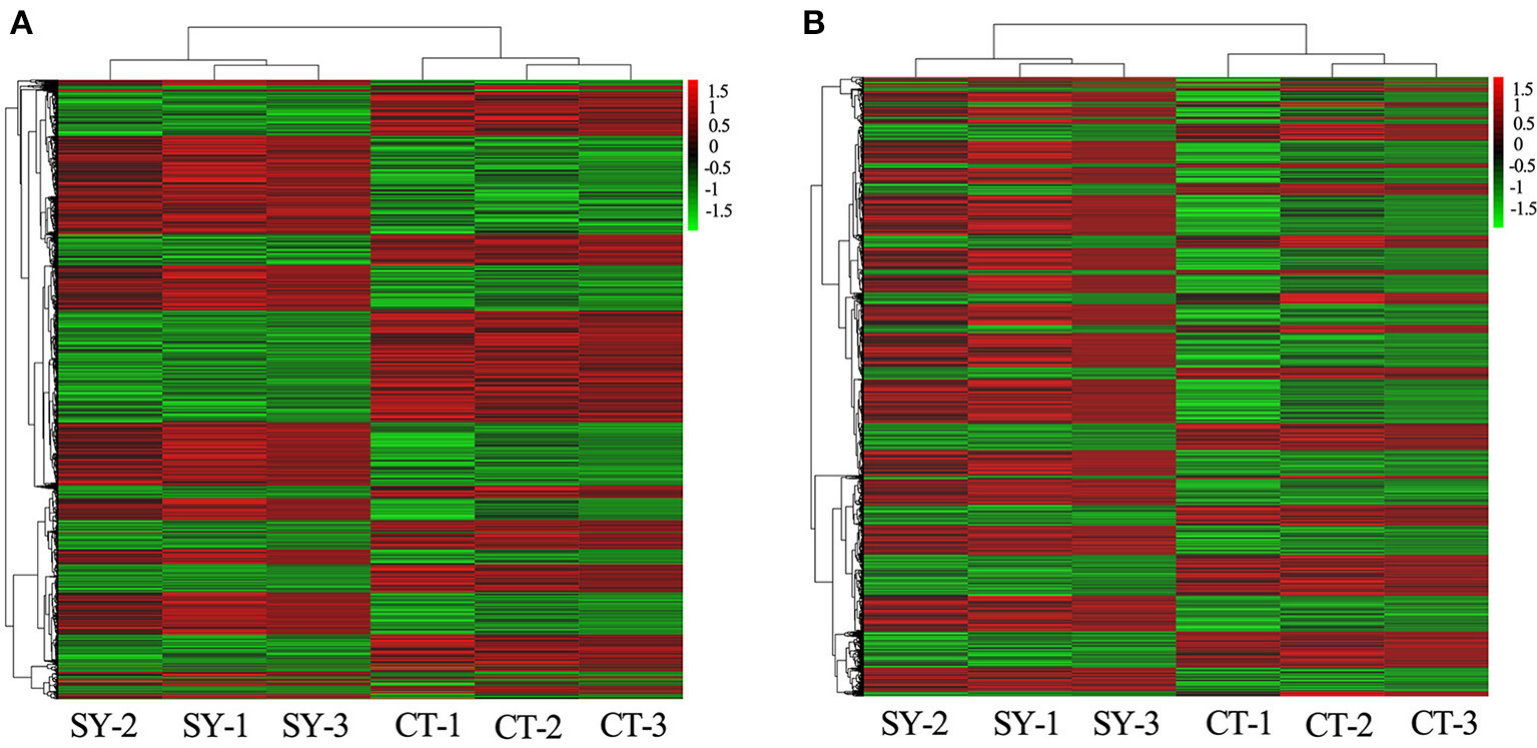
Heat maps of the differentially expressed lncRNAs and mRNAs between the healthy control (*n* = 3) and neurosyphilis groups (*n* = 3). **(A)** The dendrogram shows the relationships among the lncRNA expression profiles in the CD4+T cells acquired from neurosyphilis patients and healthy controls. **(B)** The dendrogram demonstrates the relationships among the mRNA expression profiles in the CD4+T cells acquired from neurosyphilis patients and healthy controls. “SY-” and “CT-” represent the neurosyphilis group and control group, respectively. The red block indicates up-regulated expression, and the green block indicates down-regulated expression.

Based on the expression level of the RNAs in the microarray analysis, a hierarchical clustering analysis was performed for the TOP 50 up/down-regulated lncRNAs and mRNAs, allowing us to hypothesize the relationship among these samples. The dendrogram in Figure 1A demonstrates the relationships among the lncRNA expression profiles in CD4+ T cells acquired from neurosyphilis patients and healthy controls. In terms of the mRNA expression profiles, the dendrogram is presented in Figure 1B. An excel table listing the lncRNA and mRNA microarray data is provided in Supplementary Materials 1, 2.

### Differential expression of lncRNAs and mRNAs in CD4+ T cells between the neurosyphilis and control groups

**Figure 2:**
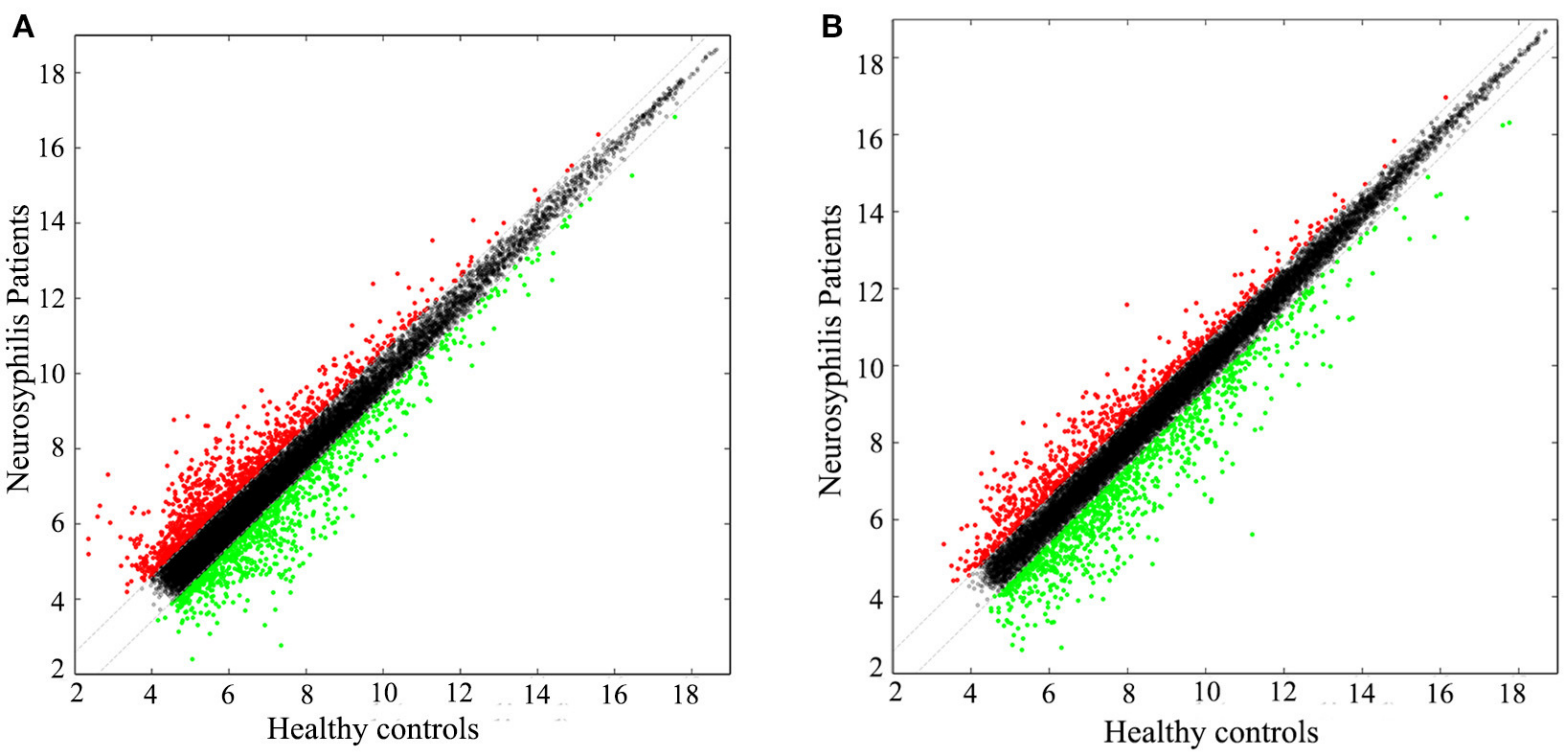
The scatter plots show the lncRNA **(A)** and mRNA **(B)** expression profiles between the groups. A total of 2258 lncRNAs and 1728 mRNAs had a fold change >1.5 and were considered over-expressed or under-expressed. The red dots indicate up-regulated expression, the green dots indicate down-regulated expression, and the black dots indicate RNAs with no significant differences between groups.

**Figure 3:**
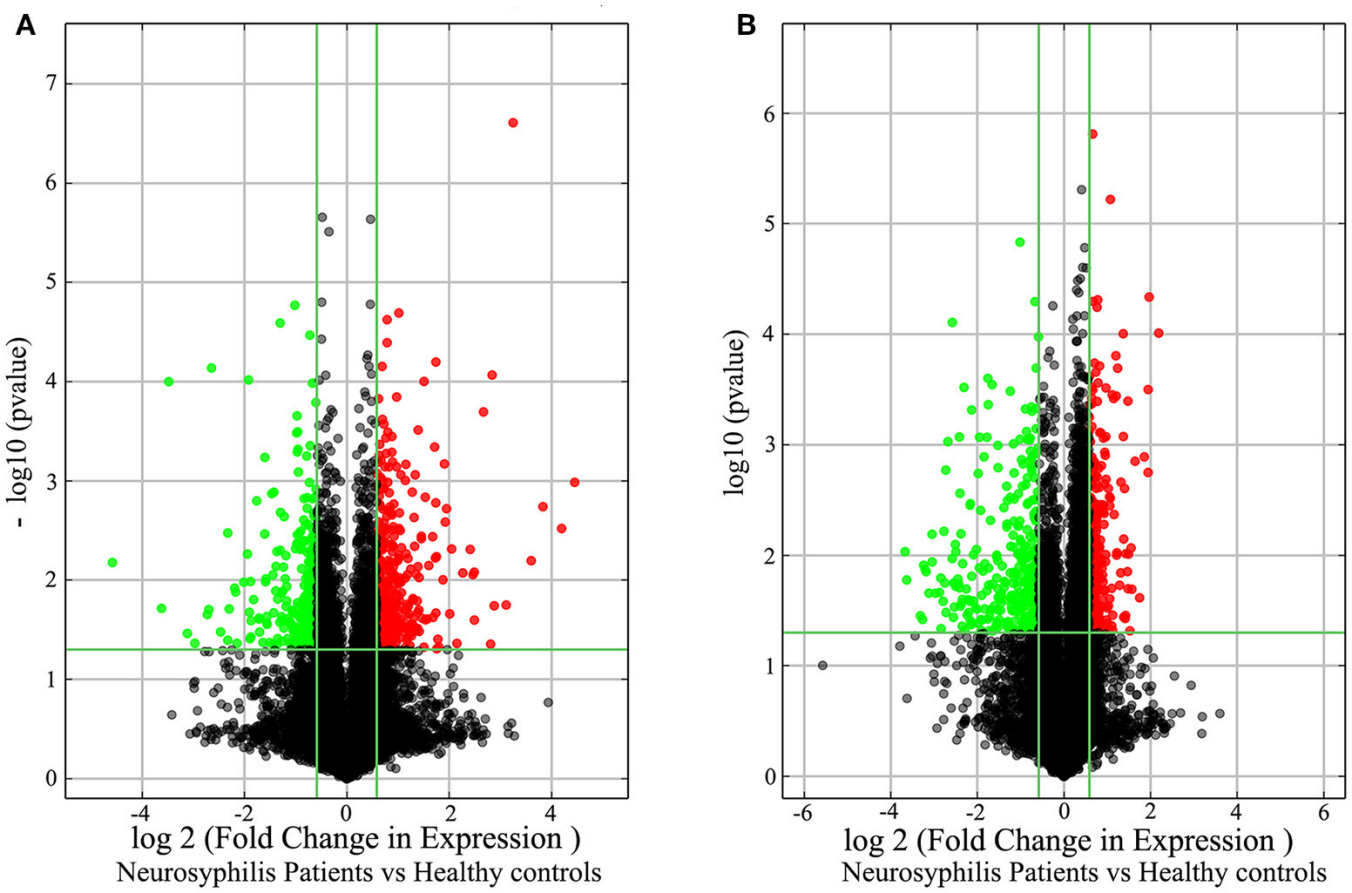
The volcano plots demonstrate the differentially expressed lncRNAs **(A)** and mRNAs **(B)** between the healthy controls and neurosyphilis patients. In total, 393 lncRNAs were significantly up-regulated, and 287 lncRNAs were significantly down-regulated in the neurosyphilis subjects. Furthermore, 287 mRNAs were significantly up-regulated, and 331 mRNAs were significantly down-regulated in the neurosyphilis subjects. The red dots indicate the RNAs with up-regulated expression, the green dots indicate the RNAs with down-regulated expression, and the black dots indicate the RNAs with no significant differences between groups. LncRNAs or mRNAs with expression fold change >1.5 and with FDR adjusted *P* < 0.05 are considered statistically significant.

In total, 17,278 lncRNAs and 15,840 mRNAs were differentially expressed between the CD4+ T cells of the three neurosyphilis patients and the three healthy control individuals. Of these transcripts, 2258 lncRNAs and 1728 mRNAs had a fold change >1.5 and were considered over-expressed or under-expressed (Figures 2A,B). Furthermore, we applied a criterion that is generally applied in microarray analyses to filter the significantly expressed RNAs. RNAs with a fold change >1.5 and a *P* < 0.05 were eligible for further analysis. Therefore, 393 lncRNAs were significantly up-regulated (fold change > 1.5, *P* < 0.05), whereas 287 lncRNAs were significantly down-regulated (fold change > 1.5, *P* < 0.05) in the neurosyphilis subjects (Figure 3A). Compared with the healthy controls, *AC068282.3* (fold change: 21.90) and *CTD-3064M3.3* (fold change: 23.93) were the most over-expressed and under-expressed lncRNAs, respectively, in the neurosyphilis patients.

After applying the criteria, 618 of 1728 differentially expressed mRNAs were selected for further analysis. Specifically, 287 mRNAs were significantly up-regulated (fold change > 1.5, *P* < 0.05), whereas 331 mRNAs were significantly down-regulated (fold change > 1.5, *P* < 0.05) in the neurosyphilis subjects (Figure 3B). *RP11-195B21.3* (fold change: 4.56) and *FOLR3* (fold change: 12.72) were the most over-expressed and under-expressed lncRNAs, respectively, in the neurosyphilis subjects compared to the healthy controls.

### Relationship between lncRNAs and their adjacent protein-coding genes

**Figure 4:**
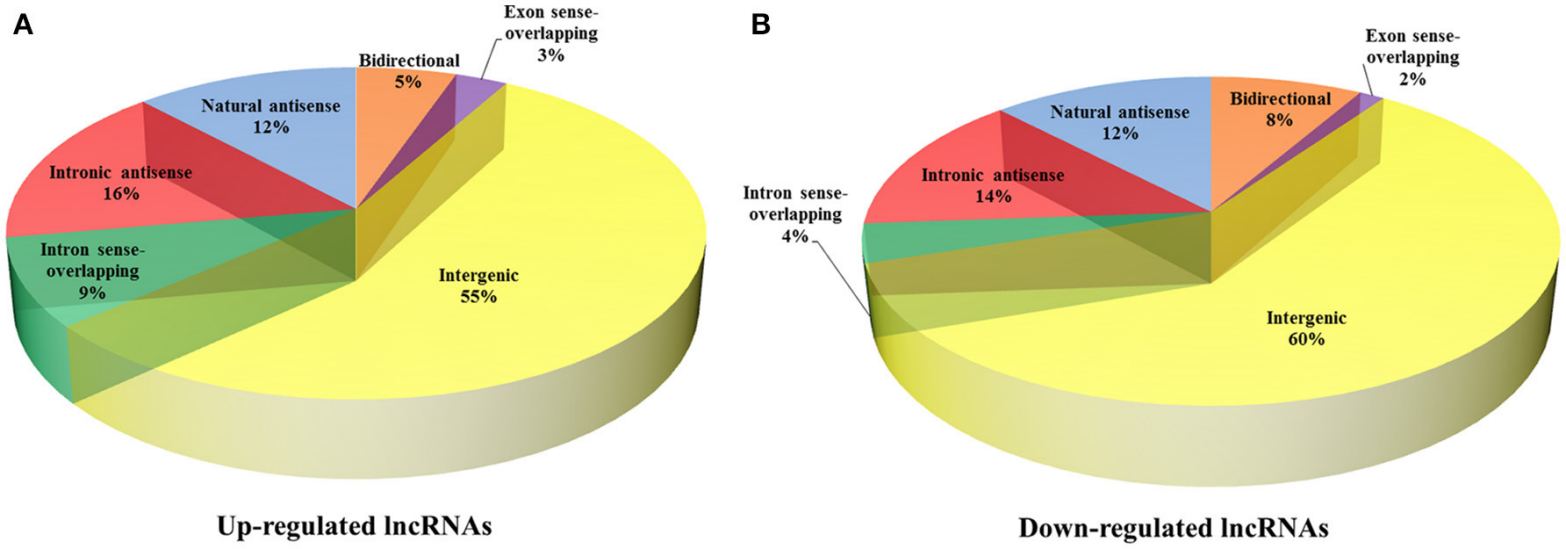
Classification of differentially expressed lncRNAs and adjacent protein-coding genes. The 393 up-regulated lncRNAs **(A)** and 287 down-regulated lncRNAs **(B)** were further classified into six classes: bidirectional lncRNAs, exon sense-overlapping lncRNAs, intergenic lncRNAs, intron sense-overlapping lncRNAs, intronic antisense lncRNAs, and natural antisense lncRNAs.

To reveal the potential roles of lncRNAs in the occurrence of neurosyphilis, the relationship between the differentially expressed lncRNAs and their adjacent protein-coding genes was analyzed. The 393 up-regulated lncRNAs and 287 down-regulated lncRNAs were further classified into six classes: bidirectional lncRNAs, exon sense-overlapping lncRNAs, intergenic lncRNAs, intron sense-overlapping lncRNAs, intronic antisense lncRNAs, and natural antisense lncRNAs. The numbers of up-regulated lncRNAs were 21, 11, 218, 34, 62, and 47, respectively, whereas the numbers of down-regulated lncRNAs were 24, 4, 173, 11, 40, and 35, respectively, in the above six classes (Table 2, Figures 4A,B). Most of the differentially expressed lncRNAs were from intergenic regions (~57.5%), intronic antisense sequences to protein-coding loci (~15.0%), and natural antisense sequences to protein-coding loci (~12.1%). The most striking finding in the analysis was the significantly altered expression of adjacent mRNA by 59 differentially expressed lncRNAs.

**Table 2:** Relationship between lncRNAs and adjacent protein-coding genes.

**LncRNA classification**	**Up-regulated expression (no.)**	**Down-regulated expression (no.)**	**Total (no.)**
Bidirectional	21	24	45
Exon sense-overlapping	11	4	15
Intergenic	218	173	391
Intron sense-overlapping	34	11	45
Intronic antisense	62	40	102
Natural antisense	47	35	82
Total (no.)	393	287	680

### Validation of the microarray results by RT-qPCR

**Figure 5:**
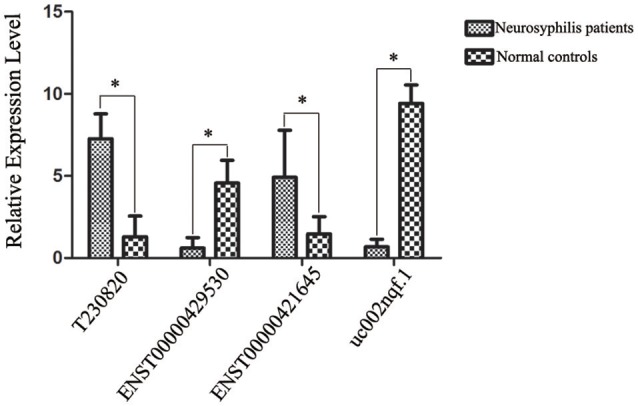
Confirmation of lncRNA expression by RT-qPCR. After normalization to β-actin expression, data were processed as the mean ± SD, and the average expression value for each lncRNA was used for the subsequent statistical analysis. Statistical analysis of the two groups was performed using Student's *t*-test. Four lncRNAs were differentially expressed between the two groups. ^*^Significant difference between the healthy controls (*n* = 23) and neurosyphilis patients (*n* = 20). A *P* < 0.05 was considered statistically significant. Each reaction was run three times, with technical triplicates for each reaction.

Based on the preliminary results of the microarray analysis, we employed both statistical and bioinformatics methods to select significant differentially expressed lncRNAs from the microarray results. *ENST00000421645, ENST00000429530, T230820*, and *uc002nqf.1* were selected for validation by RT-qPCR. The statistical analysis revealed that all four lncRNAs showed similar expression profiles to those in the results generated by the microarray analysis; *ENST00000429530* and *uc002nqf.1* were down-regulated, whereas ENST00000421645 and T230820 were up-regulated in the neurosyphilis group (Figure 5). The results were consistent with those obtained by microarray.

The four differentially expressed lncRNAs and the other 58 lncRNAs were selected for validation for their noncoding potential using two prediction software programs, CPAT and CPC. The coding potentials of *ENST00000421645, ENST00000429530, T230820*, and *uc002nqf.1* and the other 58 lncRNAs were analyzed, and the results showed that they had noncoding potential (Table 3).

**Table 3:** Sixty-two differentially expressed lncRNAs were selected for validation of their coding potential using two prediction software programs, CPAT and CPC.

**LncRNA**	**Nearby gene symbol**	**Nearby protein name**	CPC Coding potential score^*^	CPAT coding probability^*^
AK025194	SMCHD1	Structural maintenance of chromosomes flexible hinge domain containing 1	−1.250	0.012
ENST00000412639	SLC2A5	Solute carrier family 2 (facilitated glucose/fructose transporter), member 5	−0.921	0.083
ENST00000428348	LOC79999	Uncharacterized LOC79999	−0.526	0.153
ENST00000466692	NKX1-1	NK1 homeobox 1	−0.482	0.278
ENST00000564248	MAP7	Microtubule associated protein 7	−0.752	0.048
ENST00000572818	LOC79999	Uncharacterized LOC79999	−0.473	0.030
ENST00000583141	LOC79999	Uncharacterized LOC79999	−0.750	0.089
ENST00000606069	EOMES	Eomesodermin	−0.966	0.003
ENST00000606841	ADRB2	Adrenoceptor beta 2	−0.998	0.002
ENST00000608161	NKX1-1	NK1 homeobox 1	−0.973	0.008
ENST00000608243	ST6GALNAC3	ST6 (alpha-N-acetyl-neuraminyl-2,3-Beta-galactosyl-1,3)-N-acetylgalactosaminide alpha-2,6-sialyltransferase 3	−1.372	0.000
ENST00000609032	TPR	Translocated promoter region, nuclear basket protein	−1.310	0.004
ENST00000609510	AP000349.1		−0.540	0.106
GSE61474_TCONS_00156600	SMCHD1	Structural maintenance of chromosomes flexible hinge domain containing 1	−1.162	0.002
GSE61474_TCONS_00200786	ACVR1C	Activin A receptor type IC	−1.087	0.003
M18205	TCRDV2		−0.870	0.087
NR_015421	FAM115C		0.219	0.993
NR_026719	TTC3	Tetratricopeptide repeat domain 3	−1.029	0.434
NR_033972	SWAP70	SWAP switching B-cell complex 70 kDa subunit	−0.896	0.073
NR_103734	GPAT2	Glycerol-3-phosphate acyltransferase 2, mitochondrial	−0.585	0.409
NR_110549	FAM115C		3.460	1.000
T014304	COL24A1	Collagen, type XXIV, alpha 1	−1.166	0.016
T023530	ITLN1	Intelectin 1 (galactofuranose binding)	−0.994	0.050
T063868	XRRA1	X-ray radiation resistance associated 1	−0.715	0.003
T074169	DUSP16	Dual specificity phosphatase 16	−0.950	0.034
T112136	KIAA0125	KIAA0125	−0.982	0.016
T127680	MKL2	MKL/myocardin-like 2	−1.114	0.004
T230820	AP000349.1		−1.308	0.010
T239862	CCR4	Chemokine (C-C motif) receptor 4	−0.917	0.083
T245080	PROK2	Prokineticin 2	−0.954	0.007
T253607	B3GALNT1	Beta-1,3-N-acetylgalactosaminyltransferase 1 (globoside blood group)	−1.146	0.011
T268030	FAM13A	Family with sequence similarity 13 member A	−1.165	0.008
T279162	TRIO	Trio Rho guanine nucleotide exchange factor	−0.580	0.156
T296179	SLC22A23	Solute carrier family 22 member 23	−1.025	0.036
T299997	ZNF311	Zinc finger protein 311	−0.903	0.005
T321409	BBS9	Bardet-Biedl syndrome 9	−1.112	0.000
T321703	AOAH	Acyloxyacyl hydrolase	−0.811	0.016
T323320	IGFBP3	Insulin like growth factor binding protein 3	−0.788	0.240
TCONS_00008740	NKX1-1	NK1 homeobox 1	−0.732	0.052
TCONS_00012742	CRISP2	Cysteine-rich secretory protein 2	−0.575	0.026
TCONS_00014959	DUSP4	Dual specificity phosphatase 4	−0.818	0.015
TCONS_00017209	TIMM8A	Translocase of inner mitochondrial membrane 8 homolog A (yeast)	−1.099	0.031
TCONS_00017353	TIMM8A	Translocase of inner mitochondrial membrane 8 homolog A (yeast)	−1.172	0.009
TCONS_00025537	SCIMP	SLP adaptor and CSK interacting membrane protein	−1.327	0.031
TCONS_00027618	VN1R4	Vomeronasal 1 receptor 4	−1.320	0.004
ENST00000434533	AGAP10		−1.352	0.005
ENST00000455707	EPB41L5	Erythrocyte membrane protein band 4.1 like 5	−0.911	0.020
ENST00000544086	DUSP16	Dual specificity phosphatase 16	−1.300	0.007
ENST00000553061	NFE2	Nuclear factor, erythroid 2	−1.371	0.002
ENST00000562538	GSTM1	Glutathione S-transferase mu 1	-−0.677	0.089
ENST00000607735	NEFL	Neurofilament, light polypeptide	−1.229	0.001
NR_034012	WDR86	WD repeat domain 86	−0.929	0.537
NR_038966	PTPRN2	Protein tyrosine phosphatase, receptor type N2	−0.807	0.581
NR_109857	WDR86	WD repeat domain 86	−0.688	0.648
T012270	INADL	InaD-like (Drosophila)	−0.503	0.079
T133105	NLRC5	NLR family, CARD domain containing 5	−1.130	0.069
T203015	PDK1	Pyruvate dehydrogenase kinase 1	−0.943	0.002
T334257	ZC3HAV1	Zinc finger CCCH-type, antiviral 1	−0.681	0.252
ENST00000429530			−1.082	0.012
uc002nqf			−0.917	0.034
T230820	AP000349.1		−1.308	0.010
ENST00000421645	KANK1	KN motif and ankyrin repeat domains 1	−1.221	0.009

*CPC, Coding Potential Calculator; CPAT, Coding Potential Assessment Tool*.

* *Calculated by default setting*.

### lncRNA-mRNA co-expression network

**Figure 6:**
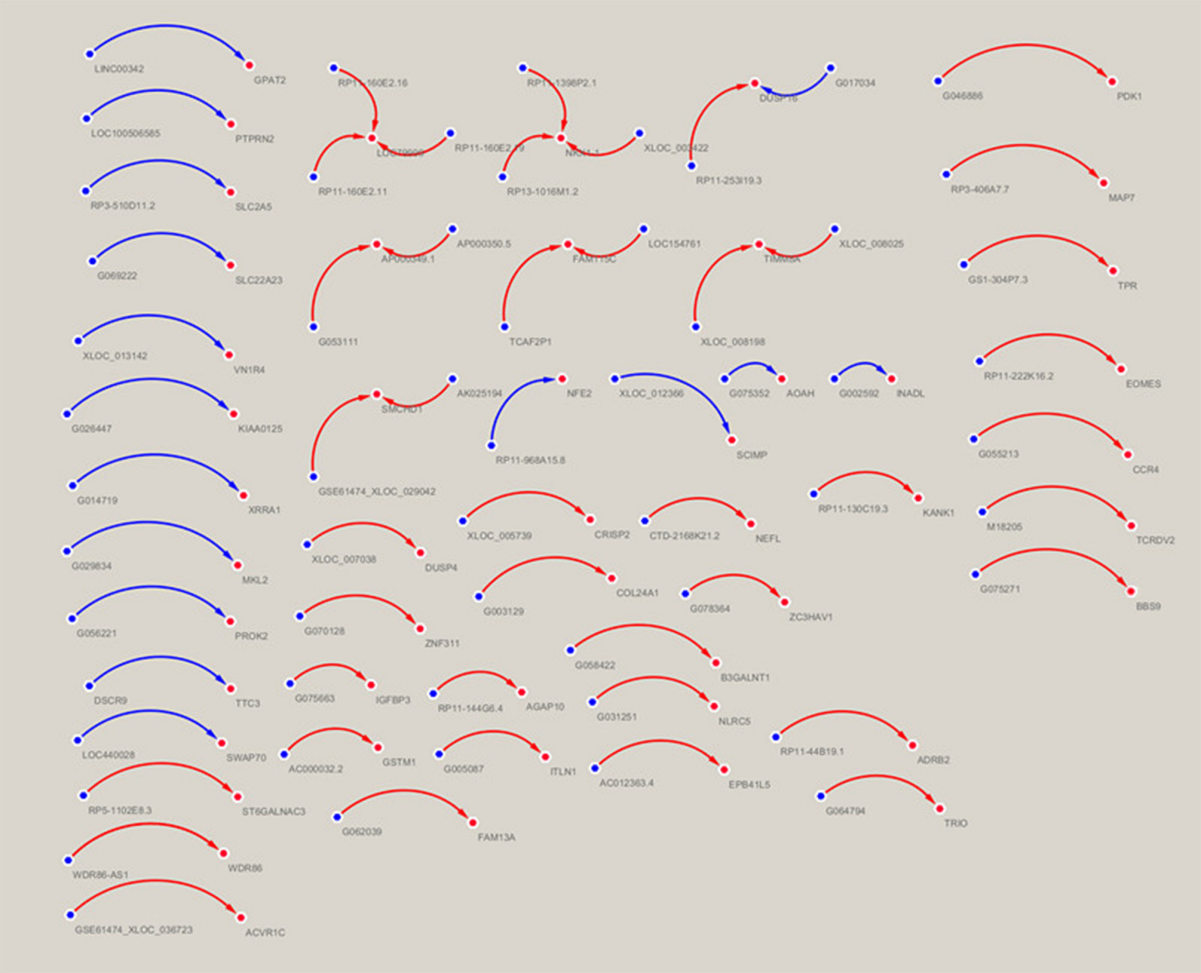
Correlation analysis between the lncRNAs and mRNAs. In the network analysis, the blue node represents the lncRNA, and the red node represents the mRNA. The blue lines indicate a negative correlation, and the red lines indicate a positive correlation.

The lncRNA-mRNA co-expression network was constructed based on the normalized signal intensities of the specifically expressed lncRNAs or mRNAs (Zhu et al., 2015). In total, 59 lncRNA-mRNA pairs of differentially expressed lncRNAs and mRNAs were identified. The network suggested that one mRNA could be correlated with more than one lncRNA. Among the 59 pairs of genes, *LOC79999* was positively correlated with lncRNAs *RP11-160E2.16, RP11-160E2.11*, and *RP11-160E2.19* and *NKX1-1* was positively correlated with lncRNAs *RP11-1398P2.1, RP11-160E2.19*, and *XLOC_003422*. The following five mRNAs were correlated with two differential lncRNAs: *DUSP16, AP000349.1, FAM115C, TIMM8A*, and *SMCHD1* (Figure 6).

The 59 differentially expressed lncRNAs in the co-expression network that had noncoding potential and were selected for validation of their coding potential by the two prediction software programs (CPAT/CPC) are presented in Table 4.

**Table 4:** The 59 differentially expressed lncRNAs in the co-expression network.

**LncRNA**	**Chromosomal location**	Regulation^*^	**Fold change**	***P*-value**
AK025194	chr18	up	1.995	0.006
ENST00000412639	chr1	up	3.368	0.006
ENST00000428348	chr17	up	1.600	0.037
ENST00000466692	chr4	down	2.723	0.009
ENST00000564248	chr6	down	1.960	0.004
ENST00000572818	chr17	up	2.061	0.003
ENST00000583141	chr17	up	2.848	0.000
ENST00000606069	chr3	down	2.548	0.015
ENST00000606841	chr5	down	5.014	0.040
ENST00000608161	chr4	down	3.035	0.001
ENST00000608243	chr1	up	1.855	0.009
ENST00000609032	chr1	up	1.506	0.030
ENST00000609510	chr22	up	6.367	0.000
GSE61474_TCONS_00156600	chr18	up	2.196	0.048
GSE61474_TCONS_00200786	chr2	up	1.743	0.000
M18205	chr14	down	2.358	0.007
NR_015421	chr7	up	2.778	0.024
NR_026719	chr21	down	1.536	0.025
NR_033972	chr11	down	1.951	0.036
NR_103734	chr2	up	1.725	0.003
NR_110549	chr7	up	2.039	0.002
T014304	chr1	down	1.514	0.029
T023530	chr1	down	2.690	0.021
T063868	chr11	down	1.532	0.001
T074169	chr12	down	1.916	0.018
T112136	chr14	up	4.443	0.043
T127680	chr16	down	1.667	0.012
T230820	chr22	up	9.512	0.000
T239862	chr3	up	1.812	0.032
T245080	chr3	up	2.505	0.005
T253607	chr3	up	1.588	0.004
T268030	chr4	up	1.575	0.036
T279162	chr5	down	3.659	0.026
T296179	chr6	down	1.510	0.016
T299997	chr6	up	2.053	0.025
T321409	chr7	up	2.613	0.031
T321703	chr7	up	1.521	0.014
T323320	chr7	up	1.826	0.019
TCONS_00008740	chr4	down	1.763	0.020
TCONS_00012742	chr6	down	1.502	0.008
TCONS_00014959	chr8	up	1.616	0.037
TCONS_00017209	chrX	down	1.592	0.008
TCONS_00017353	chrX	down	1.513	0.012
TCONS_00025537	chr17	up	1.960	0.024
TCONS_00027618	chr19	up	1.754	0.027
ENST00000421645	chr9	up	7.364	0.018
ENST00000434533	chr10	up	7.015	0.044
ENST00000455707	chr2	up	1.740	0.042
ENST00000544086	chr12	up	1.672	0.042
ENST00000553061	chr12	up	2.488	0.002
ENST00000562538	chr1	up	2.042	0.010
ENST00000607735	chr8	up	1.522	0.012
NR_034012	chr7	up	2.025	0.003
NR_038966	chr7	down	2.569	0.044
NR_109857	chr7	up	3.795	0.003
T012270	chr1	down	2.619	0.013
T133105	chr16	up	1.709	0.036
T203015	chr2	up	2.002	0.014
T334257	chr7	up	2.623	0.007

* *The annotation “up” and “down” indicate the lncRNA expression in neurosyphilis patients comparing with control subjects and the fold change demonstrates the extent of expression difference*.

### GO and KEGG pathway analysis of deregulated mRNAs

**Figure 7:**
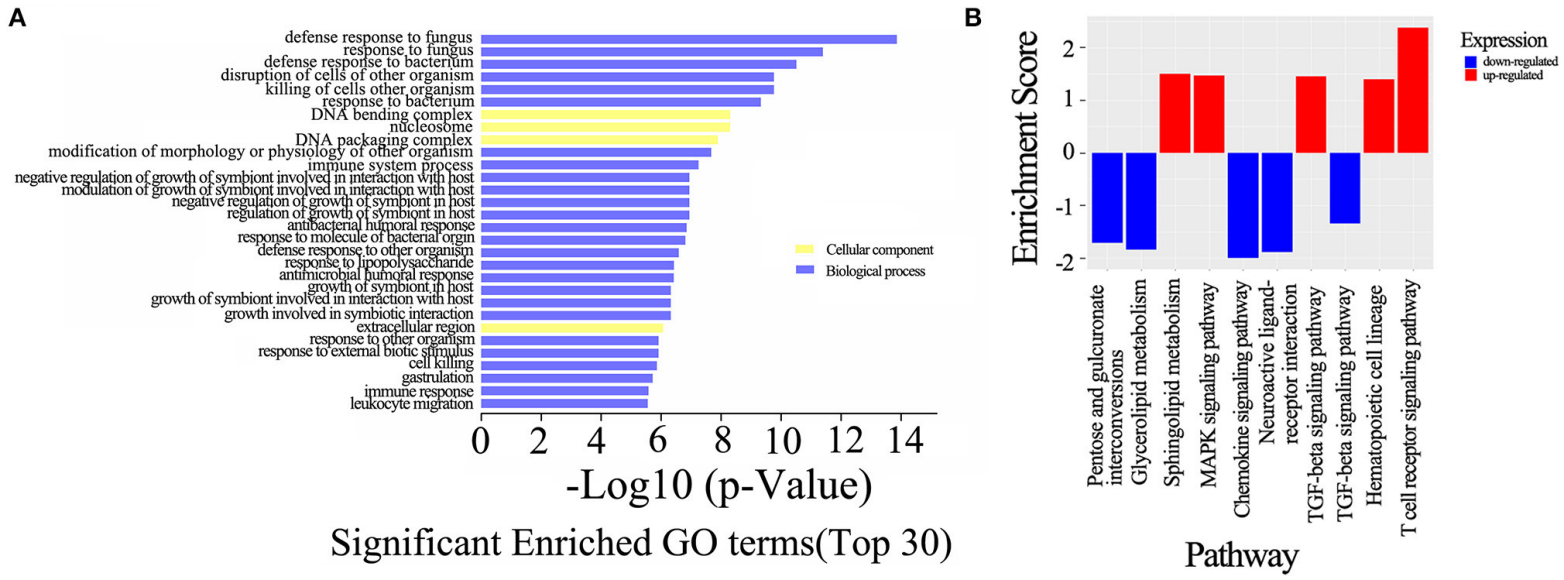
GO and KEGG pathway analysis of the dysregulated mRNAs. **(A)** The top 30 GO terms that were enriched among the differentially expressed mRNAs in the comparison of the neurosyphilis patients with the healthy controls. The enrichment score was calculated using the *P*-value; the higher the score is, the more specific the corresponding function will be. **(B)** KEGG pathway analysis of deregulated mRNAs in the comparison of the neurosyphilis patients with the healthy controls. The red column indicates up-regulated expression, and the blue column indicates down-regulated expression. The enrichment score was calculated using the *P*-value; the higher the score is, the more specific the corresponding function will be.

To evaluate the enrichment of the dysregulated mRNAs in biological processes, cellular components and molecular functions, a GO analysis was conducted, which revealed that the most significant associations were observed with the following terms: defense response to fungus, defense response to bacterium, killing of cells of other organisms and disruption of cells of other organisms. In contrast, no molecular functions were identified in the top 30 categories of the GO analysis, and only three terms involved the cellular components category: DNA bending complex, DNA packaging complex and extracellular region (Figure 7A). A subsequent pathway analysis was also conducted, and several pathways, including the T cell receptor, MAPK, and TGF-beta signaling pathways, were associated with the differentially expressed mRNAs (Figure 7B).

## Discussion

Neurosyphilis can occur at any stage following an infection with *T. pallidum* and its incidence has recently increased (Villar-Quiles and Porta-Etessam, 2016). To date, the mechanism that underlies the nervous systemic damage or inaction caused by *T. pallidum* remains unknown. In our previous study, we found that CD4+ T cells were significantly decreased in neurosyphilis patients. However, animal experiments performed by Marra confirmed that CD4+ T cells played critical roles in the immune response to the *T. pallidum* infection. Recent findings also support the notion that several lncRNAs are involved in regulating the immune response to pathogenic invasion.

To our best knowledge, the lncRNA expression profiles have not previously been described for neurosyphilis. Using NGS and cDNA library analysis, the first determination of the lncRNA expression and transcriptional signature of CD4+ T cells in response to the *T. pallidum* infection has been performed. The results suggested that transcriptional activity was lower in the neurosyphilis patients than in the healthy controls. The results obtained via qPCR validation were consistent with the microarray results. The coding potentials of *ENST00000421645, ENST00000429530, T230820*, and *uc002nqf.1* were analyzed by the CPAT and (CPC) prediction software programs, and the results showed that they had noncoding potential. Previous studies have shown that lncRNAs can be used as biomarkers for multiple infectious diseases (Van Roosbroeck et al., 2013). Therefore, the identification of several lncRNAs for use as new indicators in neurosyphilis screening is expected.

To uncover the potential roles of lncRNAs in the occurrence of neurosyphilis, we analyzed the relationship between the differentially expressed lncRNAs and their adjacent protein-coding genes. The 393 up-regulated lncRNAs and 287 down-regulated lncRNAs were further classified into six classes in accordance with their distinctive features, such as exertion of an effect on DNA, the genome location of the lncRNA, and context. The majority of differentially expressed lncRNAs were from intergenic regions (~57.5%), intronic antisense sequences to protein-coding loci (~15.0%), and natural antisense sequences to protein-coding loci (~12.1%). These observations indicate that lncRNAs may have several biological functions. For example, Kour Sukhleen *et al*. identified an *atRA-inducible*, intergenic lncRNA in different functional regions of the mammalian brain and documented its association with brain maturation and aging (Kour and Rath, 2017). Yi et al. identified differentially expressed lncRNAs in CD4+ T cells that were triggered during a latent tuberculosis infection, where most deregulated lncRNAs were from intergenic regions, natural antisense sequences to protein-coding loci, or intronic antisense sequences to protein-coding loci. Additionally, knockdown or down-regulation of specific lncRNAs can lead to decreased expression of neighboring protein-coding genes, which suggests that lncRNAs and nearby coding genes may have shared upstream regulatory or local transcriptional effects (Guttman et al., 2009; Hung et al., 2011).

To further predict the functions of the lncRNAs in CD4+ T cells that were differentially expressed in neurosyphilis patients, a GO analysis was performed with the different mRNAs that were associated with the lncRNAs in the neurosyphilis patients and healthy controls. The GO analysis results showed that *T. pallidum* stimulated CD4+ T cells and altered the expression of the lncRNAs. The differentially expressed lncRNAs were mainly involved in the biological processes. Among them, the most significant associations were observed with the following terms: defense response to fungus, defense response to bacterium, killing of cells of other organism and disruption of cells of other organism. A subsequent pathway analysis was also conducted, and only 22 pathways were associated with the differentially expressed mRNAs. The up-regulated mRNAs between the neurosyphilis patients and healthy controls were mainly involved in pathways that included the T cell receptor, MAPK, and TGF-beta signaling pathways. The mRNA expression of *CTLA4* was significantly elevated in neurosyphilis subjects according to the microarray analysis, and previous investigation has revealed that it involves in T cell receptor signaling pathway and also interacts with receptors on antigen-presenting cells and induce lymphocyte tolerance (Vasudevan et al., 2017). *IKBKB* is involved in the downstream TLR, NF kappa B and MAPK signaling pathways and plays a role in the proinflammatory immune response (Groeger et al., 2017). *TGIF1* is a transcriptional repressor which involved in T-cell proliferation and differentiation by limiting the output of the TGF-beta signaling pathway (Taniguchi et al., 2017; Wu et al., 2017). Meanwhile, the down-regulated mRNAs between neurosyphilis patients and healthy controls were mainly involved pathways, such as the TGF-beta signaling pathway and chemokine signaling pathway. Strikingly, the expression of *Smad7*, a negative regulator of the TGF-β signaling pathway, was significantly suppressed in the neurosyphilis patients. Subsequently, the TGF-β/Smad signaling pathway was activated and affected Treg/Th17 imbalance (Pang et al., 2014). Determining whether *Smad7* is associated with *T. pallidum* immune evasion requires further studies. *ARRB1* (adaptor protein beta-arrestin 1) is able to positively regulate naive and activated CD4(+) T cell survival via chemokine signaling pathway (Shi et al., 2007). Future studies that employ knockdown or overexpression techniques in a relevant model system will be necessary to investigate the potential regulatory roles of lncRNAs in neurosyphilis.

Additionally, we observed several differentially expressed mRNAs in the CD4+ T cells of neurosyphilis patients, and their biological functions were investigated. For instance, mRNA *NM_030763*, whose encoding product is the HMGN5 protein, was significantly decreased in the neurosyphilis patients. He et al. has shown that low HMGN5 expression predicts a low meningioma recurrence probability (He et al., 2015). Furthermore, down-regulation of HMGN5 inhibits IOMM-Lee and CH157 cell proliferation, enhances cell apoptosis, etc., (Kampa-Schittenhelm et al., 2015). Based on these results, we can hypothesize that *T. pallidum* inhibits the CD4+ T cell response by deregulating the HMGN5 mRNA. For the *NM_003064* mRNA, we observed decreased expression among the neurosyphilis patients. The only mRNA that was significantly elevated in the neurosyphilis patients in our investigation was *ZC3H12D*. Hong Zhang *et al*. reported that *the ZC3H12D* level was inversely correlated with the expression levels of proinflammatory genes. Their data indicated that *ZC3H12D* could suppress both the initial inflammation storm and chronic inflammation by targeting the mRNAs of cytokines and of NF-κB and c-fos (Zhang et al., 2015). We assumed that *T. pallidum* could escape immune surveillance by inhibiting NF-κB activity and IL-8 expression, which is affected by lncRNAs through down-regulation of the *NM_003064* mRNA and up-regulation of the *ZC3H12D* mRNA. As with the other differentially expressed mRNAs in our study, we also observed suppressed expression of *Hbb* in the neurosyphilis patients. The *Hba1/2* and *Hbb* mRNAs are predominantly expressed in cells of the erythroid lineage. However, recent studies, have shown HBA1/2 and HBB expression in non-erythrocyte cell types, such as neurons (Richter et al., 2009) and myocardial tissues (Son et al., 2014). Cellular expression of these hemoglobin subunits is induced in response to increased levels of intracellular reactive oxygen species (ROS), and HBA1/2 and HBB may play a protective role against oxidative stress (Liu et al., 2011; Li et al., 2013). Strikingly, the *Hbb* mRNA is also expressed in CD4+ T cells, which has not been reported in previous studies. Thus, the biological function of cellular hemoglobin in CD4+ T cells requires further elucidation. Notably, 59 lncRNAs were significantly different together with significantly different mRNAs, such as the significantly up-regulated *T230820* lncRNA (fold change = 9.512) with *ENST00000598975* mRNA (fold change = 1.636). The differentially expressed mRNAs (*n* = 195) that were isolated in this study were not identical to those published in previous reports, so further studies are necessary. Based on the genomic locations of the lncRNAs in relation to known mRNAs, the lncRNAs were predicted to be involved in gene silencing and DNA damage (Tang et al., 2014), cell proliferation (Moita et al., 2012; Yan et al., 2013), cell differentiation (Stienne et al., 2016), and cell migration and invasion (Chiyomaru et al., 2011).

The present study has some limitations. The major concern is that a control group consisted with syphilis patients without neurosyphilis was absent. Therefore, the comparison on lncRNA expression profile between neurosyphilis patients and syphilis patients without neurosyphilis was unable to conduct. Limited sample size was another disadvantage of this study. Further investigation with properly designed control groups and expanded sample size would help to elucidate the underlying mechanisms of neurosyphilis.

In summary, differential lncRNA, and mRNA expression profiles for CD4+ T cells that were acquired from neurosyphilis patients and healthy control subjects were determined, and these data provide a new direction for further research into the pathogenic mechanisms of *T. pallidum*.

## Author contributions

Conceived and designed the experiments: T-CY, J-HY, L-LL, X-YJ, JR, and L-RL. Performed the experiments: S-GZ, N-NZ, M-LT, and H-LZ. Analyzed the data: T-CY, L-LL, and YL. Contributed reagents/materials/analysis tools: X-YJ, JR, W-HZ, H-JF, and H-JL. Wrote the paper: T-CY, J-HY, and L-LL.

### Conflict of interest statement

The authors declare that the research was conducted in the absence of any commercial or financial relationships that could be construed as a potential conflict of interest.
